# Mechanisms of Long-Term Persistence of Mycoplasmas in Children with Asthma

**DOI:** 10.3390/microorganisms11071683

**Published:** 2023-06-28

**Authors:** Luisa G. Gorina, Natalya A. Krylova, Irina V. Rakovskaya, Natalia A. Geppe, Natalia A. Gamova, Olga I. Barkhatova

**Affiliations:** 1 Gamaleya National Research Center of Epidemiology and Microbiology, 123098 Moscow, Russia; 2 Department of Childhood Diseases Sechenov, First Moscow State Medical University (Sechenov University), 119435 Moscow, Russia

**Keywords:** mycoplasma infection, asthma, antigens, circulating immune complexes, DNA, microcolonies, treatment

## Abstract

Improving the management of children with asthma associated with mycoplasma infection is important. Aim: To study the duration of the persistence of antigens, and DNA in a free state, in the structures of circulating immune complexes (CICs) and living cells of *Mycoplasma pneumoniae (Mpn)* and *Mycoplasma hominis (Mh)* in children with asthma. In total, 205 children with asthma from 1 to 14 years were observed. The reaction of aggregate-hemagglutination (AHAA), the direct immunofluorescence reaction (DIF), the reaction of the polymerase chain reaction (PCR), and the culture method were used. In addition, 47 children were re-examined 1.5 months after the treatment of mycoplasma infection with azithromycin. The number of samples positive for antigens and DNA in the free state and in the structures of CICs significantly decreased. Then, 50 blood serum samples containing *Mh* antigens, and 50 samples containing *Mpn* antigens were analyzed by culture method. *Mh* was isolated in 21 (65.5%) of 32 samples containing DNA. *Mpn* was isolated from antigen-positive samples in nine cases. The presented data indicate the long-term persistence of antigens, and DNA of mycoplasma cells in the free state, in the structure of CICs, as well as in the form of “microcolonies”. A high level of CICs can be used to predict the course of the disease and the response to therapy.

## 1. Introduction

The importance of antigenemia in the pathology of human infectious diseases remains relevant at the present time. The mechanisms of the persistence of bacterial antigens in the macroorganism and the possibility of their long-term storage have not been fully studied. The influence of mycoplasma infection on the course of asthma has been shown in studies [1,2,3].

The recurrence of an obstructive syndrome in mycoplasma infection is associated with the persistence of the pathogen in the body [4,5]. Mycoplasmas can persist and multiply for a long time in the tissues of the macroorganism and change the metabolism of infected cells. Mycoplasma antigens are poorly recognized by the host body, which prevents the production of antimycoplasma antibodies and affects the persistence of the pathogen in the host body [6,7]. Prolonged persistence can cause a chronic course of the pathological process with periodic exacerbations. It has been shown that during asthma exacerbations, patients often have mycoplasma infections caused by *M. pneumoniae* and *M. hominis* [8,9,10]. The phenomenon of prolonged circulation of mycoplasma antigens in the blood of patients is noted. The long-term persistence of mycoplasmas on host cells may be due to their membrane parasitism, which allows them to escape phagocytosis [11].

Macrolides are widely used in the outpatient setting due to their high oral bioavailability. However, much attention has been paid to antibiotic resistance in the treatment of mycoplasma infections in recent years [12]. Point mutations of the macrolide-binding site located in the 23S rRNA genes or in the genes encoding ribosomal proteins L4 and L22 have been described for macrolide resistance [13,14]. Point mutations in the 16S rRNA genes have been described for tetracycline-resistant mutants of *M. pneumoniae* [14] and *M. bovis* [15].

In recent years, studies have shown the emergence of antibiotic resistance of mycoplasmas forming microcolonies [16,17]. Confocal microscopy in combination with fluorescent staining showed microcolonies attached to the abiotic surface with the biofilm architecture visible [16]. Biofilm is also involved in the emergence and phenotypic resistance of antibiotics [18]. Observation of the biofilm formation process by the *Mycoplasma pneumoniae* Fh strain on the abiotic surface by light and electron microscopy confirmed the ability of *Mpn* to form it. The ability of *Mpn* to grow as a biofilm is of great importance for understanding the causes of the long-term persistence of *Mpn* in the human body and for developing new approaches to the treatment of prolonged and chronic forms of infectious processes caused by *Mpn* [19]. Morphology and ultrastructural organization microcolonies of four species of *Mollicutes* have been described in our article [20]. The investigation was carried out using scanning and transmission electronic microscopy. Microcolonies of all species are differing radically from typical classic mycoplasma colonies. Microcolonies have a spiral form with dense centers and blades, composed of rod-shaped cells. Colonies are arranged by almost parallel rows of cells contracted closely side by side [20]. In contrast to typical colonies, microcolonies are characterized by tiny propeller-shaped colonies formed by rod-like cells tightly packed in parallel rows. This form is characterized by specific cell and colony morphology, changes in key biochemical markers, unspecific resistance to antibiotic treatments and other stresses including the immune response, and a wide spread in clinical samples. Elucidation of the role that this form might play in infectious disease might be an important step in a deeper understanding of mechanisms used by bacterial pathogens in the development of persistent infections [17].

We think that there is no complete elimination of antigens in the body since there are mechanisms in the structure of the immune system responsible for their long-term storage. To solve this problem, it was necessary to determine not only the nature but also the duration of the persistence of antigens, circulating immune complexes (CICs) containing specific antigens, the DNA of mycoplasma cells, and possibly living mycoplasma cells in patients with asthma. There is evidence of a certain role of the CICs in the pathogenesis of mycoplasma infections [21]. Mycoplasmas are one of the triggers of asthma exacerbation, and the CICs are carriers of mycoplasmas in patients with respiratory pathology [11].

In this regard, it was necessary to find out whether there is a true antigenemia, or whether living mycoplasma cells are also present in the blood of infected people. It was especially important to find out whether the DNA of mycoplasmas belonged to living cells or whether they were preserved and circulating DNA fragments.

The aim of our work was to study the duration of the persistence of antigens and DNA in a free state, in the structures of circulating immune complexes and living cells of *Mycoplasma pneumoniae* and *Mycoplasma hominis* in the blood of children with asthma.

## 2. Materials and Methods

### 2.1. Study Population

In total, 205 children with asthma were observed in the Clinic of Childhood Diseases of the Filatov Clinical Institute for Children’s Health at Sechenov University; 65 children had mild (31.7%), 121 children had moderate (59.0%), 19 children had severe (9.3%) asthma. The diagnosis was based on GINA guidelines. The children were aged from 1 to 14 years, with an average age of 6.2 ± 2.1 years (114 boys (55.6%) and 91 girls (44.4%)). The diagnosis of asthma was established on the basis of anamnestic, clinical data, general clinical, allergological, and instrumental examination. We obtained spirometry with pre- and post-bronchodilator responses in children older than 5 years. The sensitization spectrum was evaluated by determining allergen-specific IgE. All children were on basic therapy with antileukotriene drugs, non-steroidal anti-inflammatory drugs, and inhaled corticosteroids depending on the severity of asthma; however, basic therapy in children was not effective enough and all children had exacerbations of the disease.

Patients were not included in the study in the presence of decompensated conditions or acute conditions that could significantly affect the study; in the presence of concomitant urological or rheumatological pathology (since *M. hominis* can be detected in such patients); with confirmed or suspected intolerance to azithromycin.

The comparison group consisted of 60 healthy children aged 1 to 14 years, with an average age of 6.4 ± 2.3 years (34 boys (56.7%) and 26 girls (43.3%)).

Examination of children for mycoplasma infection was carried out in the laboratory of mycoplasmas and L-forms of bacteria of the Gamaleya National Research Center of Epidemiology and Microbiology, Russia.

When mycoplasma infection was detected, 47 children (main group) received treatment with azithromycin: three courses at a dose of 10 mg/kg body weight for 3 days with an interval of 4 days (taking into account the possibility of azithromycin accumulation in the affected tissues and maintaining the therapeutic concentration for 5–7 days). They were re-examined 1.5 months after treatment of mycoplasma infection. The control group consisted of 50 children not receiving treatment for mycoplasma infection (Table 1). The basic therapy did not change within three months before and after the examination.

### 2.2. Ethical Statement

The recommendations of the Declaration of Helsinki were taken into account during the research, and the protocol was approved by the Local Ethics Committee of Sechenov First Moscow State Medical University (Sechenov University). All parents and/or guardians wrote informed consent for participation in the research. Obtaining hyperimmune sera to mycoplasmas, by immunizing rabbits with mycoplasmas, was carried out in accordance with the decrees of the Ministry of Health of Russia (No753n from 26 August 2010, No774n from 31 August 2010) in the manifestation of humanity, both during the experiment itself and in relation to the maintenance, care, and daily feeding of animals. The rabbits were fed with forage enriched with environmentally friendly, fortified foodstuffs. Measures have also been taken to avoid undue stress, pain, physical suffering, and injury to the animals.

### 2.3. Bacterial Strains

The following Mycoplasma types and reference strains were used in the study: the *Mycoplasma hominis* strain H-34; the M. *Mycoplasma pneumonia* FH strain, grown in a liquid medium prepared using the method of cultivation of mycoplasmas [17].

### 2.4. Development of Hyperimmune Serum

The hyperimmune serum to antigens of the specified types of mycoplasmas was developed by rabbit immunization with mycoplasma membrane fractions that were obtained as described in [22]. In short, type Mycoplasma strains were grown in the broth for 3 days at 37 °C. Bacteria were collected at 9000 r.p.m. (13,584× *g*) for 45 min, resuspended in PBS, and disrupted with the Techpan ultrasonic disintegrator for 20 min (ten 2-min cycles with 2-min intervals). Membrane fraction was obtained by centrifugation at 40,000× *g* for 30 min. The obtained membrane fraction was washed three times with PBS and suspended in it. For intraperitoneal and intramuscular immunization of rabbits, five injections of cytoplasmic membrane preparations of mycoplasmas, purified in a stepwise sucrose gradient, with Freund’s adjuvant, were used for three months for rabbits, according to the method described in [15]. For setting up the reaction of aggregate-hemagglutination (AHAA), immune sera against mycoplasmas and ureaplasma were used with content of specific antibodies of at least 1 mg/mL.

### 2.5. Procedure for Aggregate-Haemagglutination Assays (AHAA)

The aggregate-hemagglutination reaction (AHAA) was used for detection of mycoplasma antigens in blood samples. This method was proposed for the detection of antigens of various origins by Gorina L.G and Olovnikov A.M. [23]. It is based on the use of glutaraldehyde-stabilized human erythrocytes of group 0 (1), Re (-), sensitized with aggregated high-titer specific antiserum. A feature of the method is the fact that when aggregating with a bifunctional agent, antibodies of the immune serum are introduced into three-dimensional protein complexes, as a result of which some of the active centers of the antibodies rise above the surface of the erythrocyte and become more accessible to the antigen determinants. The AHAA was carried out on V-shaped polystyrene plates; titration of blood sera was carried out on 0.2% normal rabbit serum. The sensitivity of detection of mycoplasma antigens in blood serum is 1–5 ng/cm^3^.

### 2.6. Procedure for Direct Immunofluorescence

The detection of mycoplasma antigens in the structure of the CICs isolated from the blood serum of patients was carried out by direct immunofluorescence reaction (DIF). This test was performed with ‘MycoPneumoFluoScreen’, ‘MycoHomoFluoScreen’ test systems (Niarmedic, Moscow, Russia). For direct immunofluorescence assay, detected circulating immune complexes (CICs) in blood serum were obtained on the glass, fixed by 960 ethanol; further glasses were treated with ‘MycoPneumoFluoScreen’, ‘MycoHomoFluoScreen’ test-systems [22].

### 2.7. Procedure for DNA and PCR

For mycoplasma DNA detection in blood serum and CICs, polymerase chain reaction (PCR) was used with test systems: ‘Amplisens-Mycoplasma pneumonia-Eph’; ‘AmplisensMycoplasma hominis Eph’; ‘MYC-COM-Eph’; for DNA detection *Mycoplasmaspecies* (spp.) (InterLabService, Moscow, Russia). Commercial test systems were used according to the manufacturer’s instructions.

### 2.8. Procedure for Detection of CICs

CICs were detected in blood serum by precipitation with 3.5% polyethylene glycol (6000 Da) [22]. The AHAA was carried out on V-shaped polystyrene plates; titration of blood sera was carried out on 0.2% normal rabbit serum. The sensitivity of detection of mycoplasma antigens in blood serum is 1–5 ng/cm^3^.

### 2.9. Cultural Method

Commercial nutrient mediums, “Difco PPLO Broth” and “Difco PPLO Agar”, with the addition of 20% horse blood serum, penicillin (1000 units/mL), phenol red growth indicator (0.005%), and SP-4 medium recommended for isolation of hard-to-cultivate mycoplasma species were used to isolate mycoplasmas from blood serum and CICs [24].

The species identity of the isolated “microcolonies” was confirmed in Immunofluorescence reaction (RIF), classical PCR, and real-time PCR (RT-PCR). For this purpose, flushes made with saline solution from plates with “microcolonies” of different passages were examined using reagents of the scientific production company “Syntol” for the qualitative and quantitative determination of mycoplasmas. The analysis was carried out according to the instructions (version 230408 ANK).

### 2.10. Statistical Analysis

“Medico-biological statistics. BIOSTATISTICS programs for WINDOWS and DOS IBM-PC“ (StatSoft Inc., Tulsa, OK, USA) was used to analyze the results. Statistical significance was assumed at *p* < 0.05.

## 3. Results and Discussion

### 3.1. Comprehensive Approach to Laboratory Diagnosis of Mycoplasma Infection in Children with Asthma

In 205 patients with asthma, *M. pneumoniae* (*Mpn*) and *M. hominis* (*Mh*) antigens in the free state were found in AHAA in 149 (72.7%) and 102 (49.8%) of cases, respectively. There was no significant difference when comparing the number of detected mycoplasma antigens in children with varying degrees of severity of asthma. In healthy children of the comparison group, *Mpn* and *Mh* antigens were detected via AHAA much less frequently: *Mpn*—in five (8.3%) children (*p* < 0.001); *Mh*—in three (5.0%) children (*p* < 0.001).

In the study of blood serum samples of children with asthma, DNA of *Mpn* in serum (PCR) was found in 15 (7.3%), and *M. hominis* in 34 (16.6%) of cases. Antigens of *Mpn* and *Mh* were detected in AHAA in the blood of patients significantly more frequently than DNA by PCR (*p* < 0.01). Specific CICs isolated from patients’ blood sera contained both antigens and DNA from *Mpn* and *Mh* cells. *Mpn* antigens were found in the structure of CICs (DIF) in 138 (67.3%), *Mh* antigens—in 95 (46.3%) of cases; DNA in CICs (PCR)—in 55 (26.8%) and 84 (41.0%) of cases, respectively. DNA in the structure of CICs was detected more often than in the free state. The results are significant. The data obtained indicate that in some patients, *Mpn* and *Mh* antigens can be in the blood not only in the form of soluble molecular compounds in a free state but also in the structure of CICs.

The persistence of mycoplasma antigens in immune complexes is often observed in mycoplasma infections [11]. The determination of specific antigens and DNA in a free state and in the structure of CICs is shown to determine the tactics of management of patients with long-term antigenemia and to improve the methods of controlling the therapy of mycoplasma infection.

### 3.2. Treatment Effectiveness of Mycoplasma Infection in Children with Asthma

**Figure 1:**
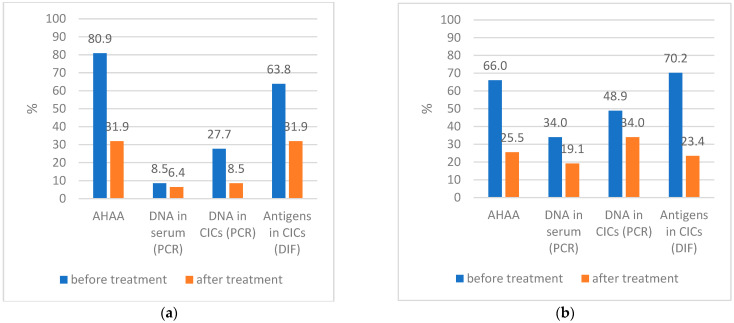
(**a**) Frequency of detection of *Mycoplasma pneumoniae* antigens and DNA in patients before and after treatment; (**b**) Frequency of detection of *Mycoplasma hominis* antigens and DNA in patients before and after treatment.

A total of 47 children were re-examined 1.5 months after treatment of mycoplasma infection. The frequency of detection of mycoplasma DNA and antigens in patients before and after treatment is shown in Figure 1a (*Mpn*) and in Figure 1b (*Mh*).

There was a significant decrease in the detection of *Mpn* antigens in the AHAA (*p* < 0.001), in CICs by DIF (*p* < 0.001), and in the indicators of specific DNA in the CICs by PCR after treatment (*p* < 0.001). Low values of DNA *Mpn* in blood sera indicate the difficulty of determining mycoplasmas in the blood. There was also a significant decrease in the detection of *Mh* antigens in the AHAA (*p* < 0.001), in CICs by DIF (*p* < 0.001), in the indicators of specific DNA in serum by PCR (*p* < 0.05), and in CICs by PCR after treatment (*p* < 0.05); thus, the number of samples positive for antigens and DNA in the free state and in the structure of CICs significantly decreased after treatment. According to our data, DNA detection in the structure of CICs is a more informative method than DNA detection in blood serum. However, some children had incomplete elimination of antigens and DNA of *Mpn* and *Mh* cells, which requires continued therapy.

We also analyzed the number of exacerbations of asthma in patients. The basic therapy did not change within three months before the start of therapy for mycoplasma infection with azithromycin and during the same period after the end of the treatment. After the course of treatment for mycoplasma infection, the number of exacerbations of asthma within three months decreased by 2.3 times compared to the same period before treatment, and the prescription of bronchodilators also decreased. There were an average of 2.8 ± 0.9 exacerbations of asthma per patient ((M ± SD) where M is the mean value, SD is the standard deviation) within three months before the start of therapy for mycoplasma infection, and 1.2 ± 0.5 exacerbations of the disease (*p* < 0.001) during the same period of time after the end of treatment for mycoplasma infection.

In the control group (50 children with asthma associated with mycoplasma infection who were not treated for mycoplasma infection) the number of exacerbations of asthma three months after the examination did not change significantly. Within three months before the examination for mycoplasma infection, there were an average of 2.7 ± 0.6 exacerbations of bronchial asthma per patient, during the same period after the examination −2.5 ± 0.6 disease exacerbations (*p* = 0.09, *p* > 0.05).

The tolerance of azithromycin was good.

This addition to the complex treatment of asthma associated with mycoplasma infection, along with the basic broncholytic therapy of macrolides, improves the course and prognosis of the disease. A comprehensive approach to the laboratory diagnosis of mycoplasma infection increases the effectiveness of monitoring the treatment of mycoplasma infection and improves the prognosis of asthma in children.

It is also important to study the problem of non-specific resistance to antibiotics in some children.

### 3.3. The Results Obtained with the Cultural Method

Earlier from seven CICs samples precipitated from the serum of blood containing *M. hominis* DNA and from two CICs samples containing *M. pneumoniae* DNA, atypical cultures of “microcolonies” of *M. hominis* and *M. pneumoniae* were isolated; their specificity was confirmed not only by DIF and PCR but also by the ability to grow on a solid medium for mycoplasmas. As previously shown, the cells of “microcolonies” are resistant to various adverse factors that cause the death of cells of typical mycoplasma colonies, most likely due to changes in their physiological characteristics and their complete antibiotic resistance [17,25,26,27].

A qualitative molecular genetic method for detecting the DNA of microorganisms of the genus Mycoplasma in the test material was used as an additional study. For this purpose, CICs isolated from the blood serum of 53 children with asthma were examined for the presence of the DNA of mycoplasma. DNA of *Mycoplasma* spp. was detected in 31 samples, which additionally confirms the persistence of DNA in the structure of CICs; thus, CICs seem to be a kind of depot not only for antigens and DNA fragments but also for maintaining viable mycoplasma cells.

In our work, blood serum samples were sown on nutrient media for a more complete answer to the question of whether these cellular components of mycoplasmas belong to living cells, or only antigenemia, and preservation of DNA fragments take place. In total, 50 blood serum samples containing *M. hominis* antigens and 50 samples containing *M. pneumoniae* antigens were analyzed by culture method. It was found that 32 out of 50 samples contained DNA of *M. hominis*, and 18 samples did not contain DNA. *M. hominis* was isolated by culture method in 21 (65.5%) of 32 samples containing DNA of *M. hominis*. *M. pneumoniae* was isolated from antigen-positive samples only in nine cases.

Subsequently, only PCR-positive serum samples were used for culture isolation.

Based on the obtained results, it can be concluded that the blood of infected patients may contain not only antigens and DNA of cells in a free state and in the structure of CICs but also living mycoplasma cells.

It has been shown that colonies isolated from the blood and CICs of infected patients differ from typical mycoplasma colonies. These colonies, called “microcolonies”, grow much more slowly than classical colonies and interlace with great difficulty, although sometimes they form a solid lawn. The identity of the isolated mycoplasmas was shown using a set of methods. It should be noted that typical colonies have never been isolated from blood samples and CICs. There is also the problem of elimination of “microcells” of mycoplasmas, which have absolute resistance to antibiotics and to various adverse factors to which cells of typical colonies are sensitive; therefore, the cells of “microcolonies” have significant advantages in comparison with the cells of typical colonies. Despite the small size of the colonies, their cells have greater reproduction energy; the number of “microcolonies” in one population is 3.5–4 times higher than the number of typical colonies [17].

This problem is one of the main tasks, the solution of which will increase the effectiveness of etiotropic therapy for asthma associated with mycoplasma infection.

## 4. Conclusions

The presented data indicate the long-term persistence of antigens and DNA of mycoplasma cells in the free state, in the structure of CICs, as well as in the form of “microcolonies” in the blood of children with asthma. Identification of specific CICs and differentiation of their composition provides valuable information about the dynamics of their formation and about the presence of a link between their long-term persistence with the duration and severity of the disease. In this regard, the long-term presence of specific CICs in the body may have diagnostic significance. Mycoplasmas can be considered one of the factors inducing exacerbations of asthma in children. Patients with asthma are shown to conduct a dynamic examination for mycoplasma infection. In addition, a high level of CICs can be regarded as a risk factor for relapse and these data can be used to predict the course of the disease and the response to etiotropic therapy.

## Figures and Tables

**Table 1:** Characteristics of the enrolled patients in main and control groups.

	Main Group *n* = 47 (Children Treated for Mycoplasma Infection) 1–14 Years	Control Group *n* = 50 (Children Not Treated for Mycoplasma Infection) 1–14 Years
Age, years (SD)	6.2 (2.3)	6.3 (2.4)
Gender of the children, *n* (%)		
Boys	27 (57.4)	29 (58.0)
Girls	20 (42.6)	21 (42.0)
Children with mild asthma, *n* (%)	15 (31.9)	16 (32.0)
Children with moderate asthma, *n* (%)	27 (57.4)	28 (56.0)
Children with severe asthma, *n* (%)	6 (12.0)	5 (10.6)
Duration of disease, years (SD)	2.1 (0.7)	2.2 (0.8)
Number of children aged 5–14 who underwent spirography and airflow reversibility testing, *n* (%)	27 (57.4)	26 (52.0)
The number of exacerbations of asthma per patient during the three months before the examination (SD)	2.8 (0.9)	2.7 (0.6)

## Data Availability

Not applicable.
